# Lysosomal autophagy promotes recovery in rats with acute knee injury through TFEB mediation

**DOI:** 10.1186/s13018-020-1573-3

**Published:** 2020-02-21

**Authors:** Qingquan Xia, Xuhua Wu, Ke Rong, Zhenyu Zhou, Xujun Li, Teng Fei, Xiaofan Yin

**Affiliations:** Department of Orthopedic, Minhang Hospital, Fudan University, No.39 Xinling Road, Minhang District, Shanghai, 201100 China

**Keywords:** Acute knee injury, Lysosome, Autophagy, TFEB gene

## Abstract

**Background:**

To study the role of lysosomal decomposition and elimination of old bone matrix, as well as the mechanism of promoting chondrocyte growth and bone recovery through the perspective of TFEB-mediated lysosomal autophagy.

**Methods:**

Rat models of acute knee injury were designed, and autophagy flow was detected by injection of autophagy inhibitors 3-methyladenine. Autophagy flow was detected by RFP-GFP-LC3 double fluorescence molecule. The expression of TFEB, DRAM, MAPLC3, and MITF were analyzed by Western blot, and the expression of genes NITF, Bcl2, and TYR in rat cartilage tissues were detected by RT-PCR.

**Results:**

The number of autophagosomes was increasing in the auto group compared with the inhibitor-auto group and normal group. There was a significant difference of LC3 levels in the auto group and inhibitor-auto group compared with the normal control. The expression of TFEB, DRAM, MAPLC3, and MITF proteins by Western blot analysis were significantly increased in the auto group and decreased in the inhibitor-auto group. The expression of NITF, Bcl2, and TYR by RT-PCR determination were higher in the auto group and inhibitor-auto group than the normal group.

**Conclusions:**

Autophagy can inhibit apoptosis, promote chondrocyte growth and bone regeneration, and restore knee joint injury of rats. The main mechanism is to promote the effect of TFEB-mediated lysosomal autophagy.

## Introduction

Lysosomes are organelles that decompose proteins, nucleic acids, polysaccharides, and other biological macromolecules [1]. They are membranous organelles in protozoa and multicellular animal cells, while bacteria do not have lysosomes [2]. Autophagy is a conservative evolutionary process in which cell proteins and organelles are engulfed by autophagosomes and eventually transferred to lysosomes for degradation [3]. Autophagy is not only an important nutrient supply pathway for eukaryotic cells, but also an important degradation mechanism for cell clearance of necrotic organelles and proteins and inhibition of inflammatory body activation [4]. At present, most researches on autophagy focus on the early stage of autophagy, and most autophagy-related genes are found to regulate the formation of autophagosomes, but relatively few researches on lysosomes in autophagy.

TFEB (transcription factor EB) is discovered in recent years [5] and is a novel signal transcription regulator promoting autophagy. TFEB is isolated in the cytoplasm and transfers to the nucleus for coordinating the expression and regulation of lysosomes. TFEB is considered the main activating agent for lysosomal autophagy gene transcription [6]. TFEB-mediated lysosomal autophagy is involved in many diseases but has not been reported in promoting the recovery of acute knee injuries. This research intends to study the role of lysosomal decomposition and elimination of old bone matrix, as well as the mechanism of promoting chondrocyte growth and bone recovery through the perspective of TFEB-mediated lysosomal autophagy.

## Experiment

### Materials

The materials used are as follows: autophagy inhibitor 3-methyladenine (3-MA), PBS (0.1 M, pH 7.0), 2.5% glutaraldehyde, pancreatic enzyme, TRIzol RNA agent, DMEM/1640, FBS (Invitrogen), cell culture dish 6/12/48 plate (Thermo Fisher), sterile pipette 5 mL/10 mL (Costar, USA), liquid transfer gun 10 μL/20 μL/200 μL/1000 μL (Eppendorf), centrifuge tube 10 mL/50 mL (Thermo Fisher), PCR instrument: conventional PCR instrument and Western blotting instruments (Eppendorf), confocal microscope (Olympus, Germany), flow meter BD, ultra-thin slicer (LKB-V, JEOL Co. Japan), and transmission electron microscope (Jem-2000EX, JEOL Co. Japan).

### Methods

#### Rat model of acute knee injury [7]

The improving Hulth method was adopted. In anesthetized rats, the right lower extremity skin was taken to sterilize the knee joint under aseptic conditions. The medial incision was taken to expose the knee joint, and the anterior and posterior cruciate ligaments and medial collateral ligaments were cut off. Two thirds of the medial meniscus was removed, and the articular surface was retained. The articular cavity was closed layer by layer, and the affected limb was not fixed. For 2 weeks, the model rats were divided into two groups (group B and group C), in which group C rats were injected with an autophagy inhibitor 3-methyladenine (3-MA) in the joint space of the knee joint. Meanwhile, the control group of healthy and normal rats was set and numbered as group A.

#### Morphological characteristics of articular cartilage in rats

The articular cartilage tissues of rats in groups A, B, and C were taken out respectively. Chondrocytes were extracted and washed with PBS (0.1 M, pH 7.0). The cells were digested with trypsin, washed, centrifuged, and fixed with 2.5% glutaraldehyde overnight at 4 °C. Subsequently, the cells were treated with osmium tetroxide for 30 min and then fixed to dehydrate with 50~100% ethanol of 10% gradient. Ultra-thin sections were prepared using an ultra-thin slicing mechanism, then stained with uranium dioxyacetate and lead citrate, and observed with a transmission electron microscope.

#### Detection of autophagy flow by RFP-GFP-LC3 [8]

The cartilage tissue cells were digested into single cells with trypsin and seeded in 48-well plates at a cell density of 5 × 10^4^/well. When the cell density grew to 40%, 0.5 μL adenovirus carrying RFP-GFP-LC3 molecule was added for transfection. The transfection efficiency reached the maximum value at 36–48 h. PBS was washed for three times, and 4% paraformaldehyde was added and fixed for half an hour, then DAPI staining was performed. The anti-quenching agent was sealed and observed by laser confocal microscope.

#### Western blot analysis of TFEB, DRAM, MAPLC3, and MITF expression [9]

Total proteins of tissue were extracted from the rat, and 20 μg proteins were sampled. Five percent concentrated gel and 12% isolated gel were prepared respectively to isolate proteins by SDS-PAGE. Objective and internal reference proteins were transferred to the NC membrane and then closed with 5% skimmed milk powder sealing fluid for 2 h at room temperature. Rabbit anti-human primary antibody TFEB (1:500), rat anti-human primary antibody DRAM (1:500), rat anti-human primary antibody MAPLC3 (1:500), rat anti-human primary antibody MITFEPB41 (1:500), and rat anti-human primary antibody β-actin (1:1000) were added and incubated at 4 °C overnight. TBST was washed four times, then HRP-labeled sheep anti-rat secondary antibody (1:5000) was added and incubated at 37 °C for 1 h. TBST was washed four times. Color was developed with ECL luminescent solution, protein bands were exposed by gel image analysis system, and images were photographed and quantitatively analyzed. The experiment was repeated three times.

#### Expression of NITF, Bcl2, and TYR

The operation is carried out according to the following instructions: the method of TRIzol was used to extract whole RNA. Nucleic acid protein complex was isolated by TRIzol RNA agent. Nucleic acid protein complex was extracted by chloroform and precipitated in isopropanol. The complex was cleaned by 75% ethanol and purified by RNase-free water. The expression level of GAPDH was selected as internal reference and reversed total RNA according to the iScript cDNA synthesis kit (Bio-Rad Laboratories, Hercules, CA). Primers for PCR detection were designed and synthesized according to the information of target gene sequences as shown in Table 1. The amplifications were performed in a 96-well plate at 95 °C for 10 min, followed by 40 cycles of 95 °C for 15 s and 60 °C for 1 min. Each sample was run in triplicate. The relative miRNA-126 and mRNA expression was expressed using the 2^−ΔΔCt^ method.

**Table 1:** Primer sequence list

Primer name	Primer sequence	Primer size (bp)
GAPDH-F	CCTGGAGAAACCTGCCAAGTA	201
GAPDH-R	TCATACCAGGAAATGAGCTTGAC	
NITF-F	ACTGTTCTGGGGGTTTGCG	200
NITF-R	CTGGGTTTTGATGGAATAGGAG	
Bcl2-F	ATTATAGAGCGATACAAGGGGG	202
Bcl2-R	TCTCGTACACTTCGGAGATGG	
TYR-F	TGCGGCAGGCTCTATCCAGAGG	201
TYR-R	CCACTGCCACCGACAGCGTC	

#### Apoptosis was detected using flow cytometry [10]

The cells in logarithmic growth phase were inoculated in 6-well plates and cultured for 24 h. The medium was sucked away. After incubating in each hole with cultured medium for 24 h, the cells were collected and fixed overnight with precooling 70% ethanol. Next day, the cells were resuspended in 40-μL phosphate-citrate buffer solution, stained at 4 °C for 30 min, and the apoptosis was detected by flow cytometry.

### Statistical analysis

All experiments were replicated independently at least three times. The data were analyzed using one-way analysis of variance (ANOVA) and are presented as the mean ± standard deviation (SD). Statistical significance was defined as *p* < 0.05.

## Results

### Morphological characteristics of articular cartilage in rats

**Fig. 1:**
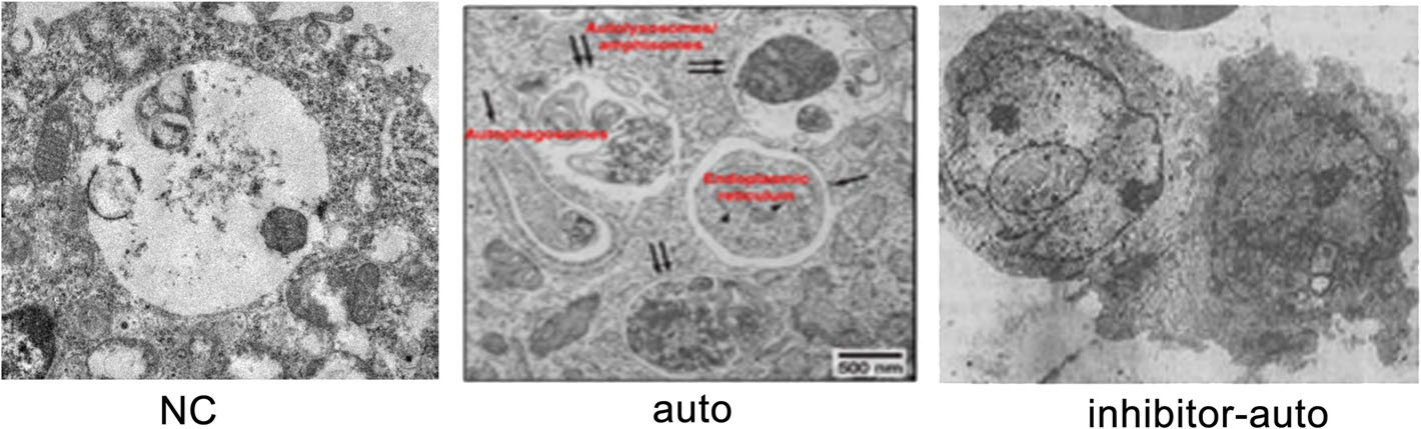
Autophagosome morphology of articular cartilage in rats of different groups. The black arrow indicated the autophagosomes in articular cartilage in rats

The number of autophagosomes was increasing in the auto group compared with the inhibitor-auto group and normal group, while the number of inhibitor-auto group declined after using auto-inhibitor agent (Fig. 1).

### Detection of autophagy flow by RFP-GFP-LC3

**Fig. 2:**
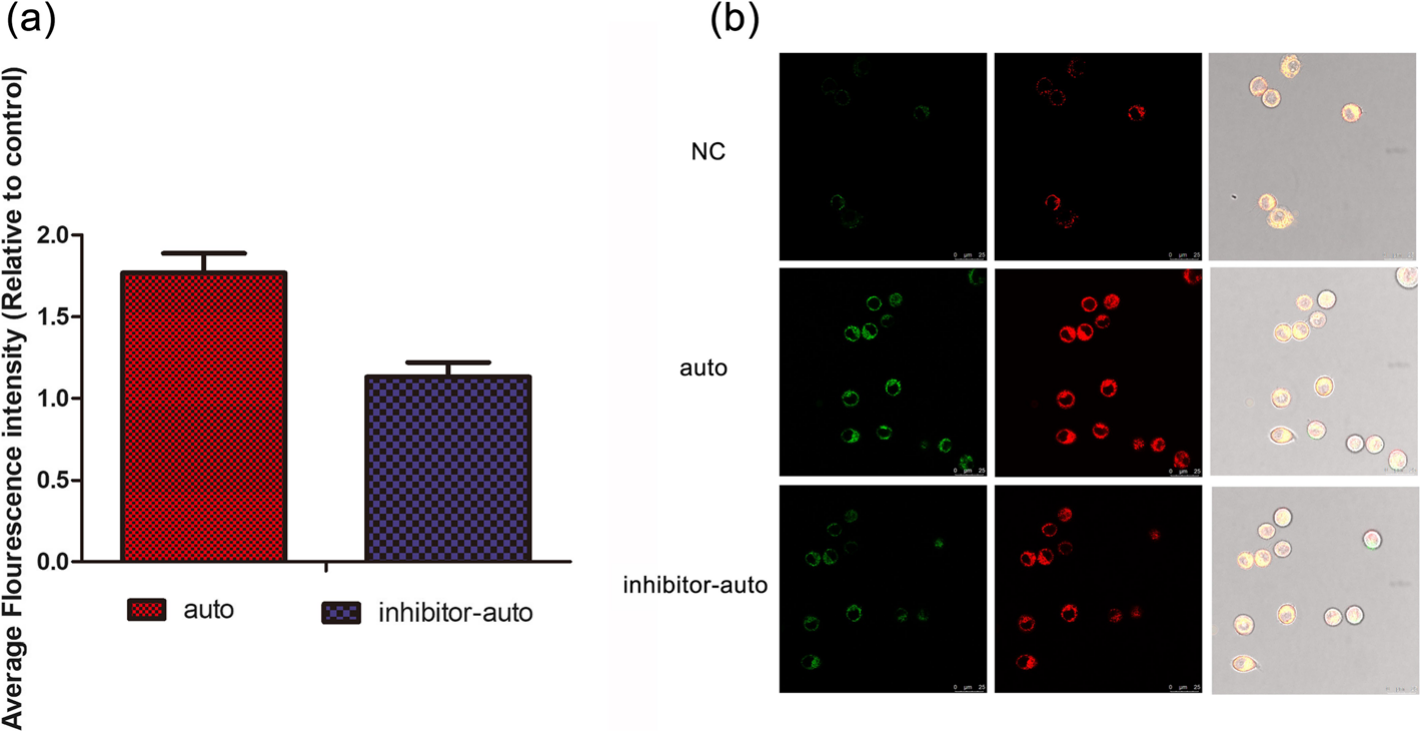
Detection of autophagy system by RFP-GFP-LC3 in rat cartilage tissue. (mean ± SD, *n* = 3). **a** Average fluorescence intensity (relative to control). **b** Confocal imaging

Increased autophagosome formation or decreased autophagic degradation will lead to LC3 levels increase. Therefore, LC3 transformation needs to be detected. The results showed that there was a significant difference of LC3 levels in the auto group and inhibitor-auto group compared with the normal control (Fig. 2a). In order to more intuitively observe the autophagy in cells, the autophagy was observed under the laser confocal microscope. The results showed that the fusion of autophagy and lysosome in the control group significantly reduced the green fluorescence signal of RFP-GFP-LC3 molecule (Fig. 2b).

### Western blot analysis of TFEB, DRAM, MAPLC3, and MITF expression

**Fig. 3:**
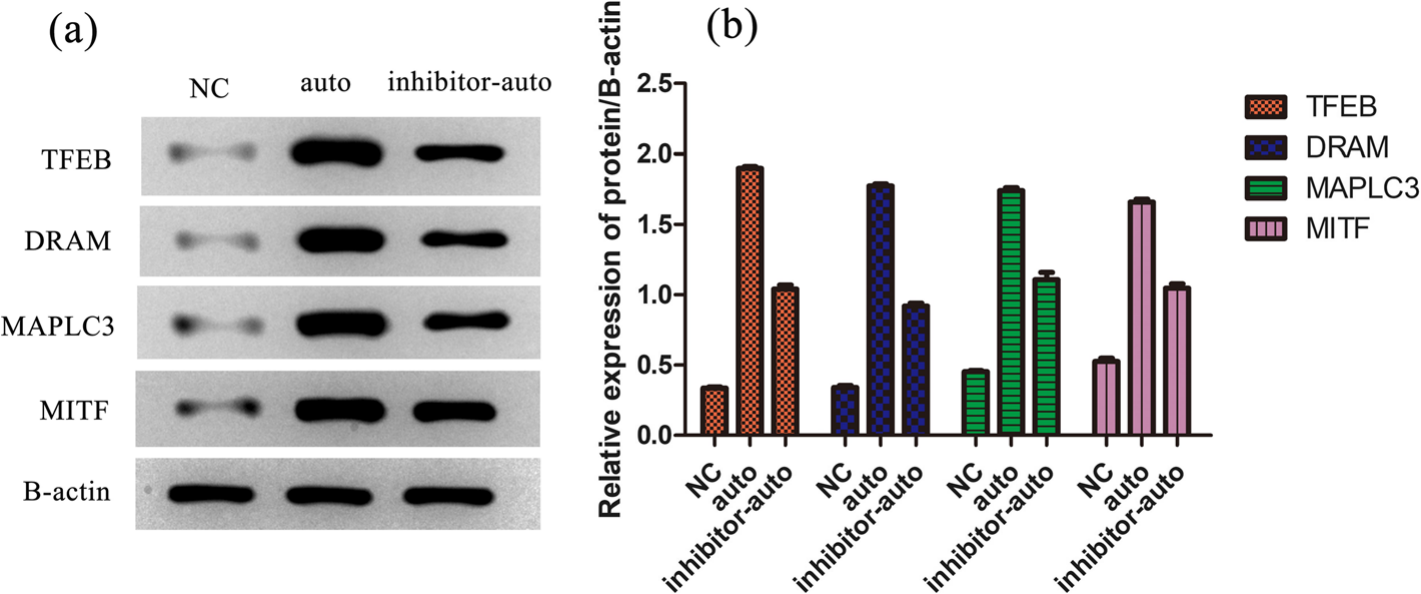
Western blot analysis of protein level of TFEB, DRAM, MAPLC3, and MITF in different groups determined by Western blotting (mean ± SD, *n* = 3). **a** Grayscale. **b** Relative expression of protein/β-actin

The expression of TFEB, DRAM, MAPLC3, and MITF proteins were significantly increased in both the auto and inhibitor-auto groups compared with the normal group. Protein expression level of the inhibitor-auto group was decreased compared with the auto group. This indicated that autophagy response was partially inhibited. The expression of autophagy signature protein MAPLC3 was significantly increased (Fig. 3a). MAPLC3 is a marker protein of autophagosome, and its expression level can reflect the level of autophagy.

### Expression of NITF, Bcl2, and TYR

**Fig. 4:**
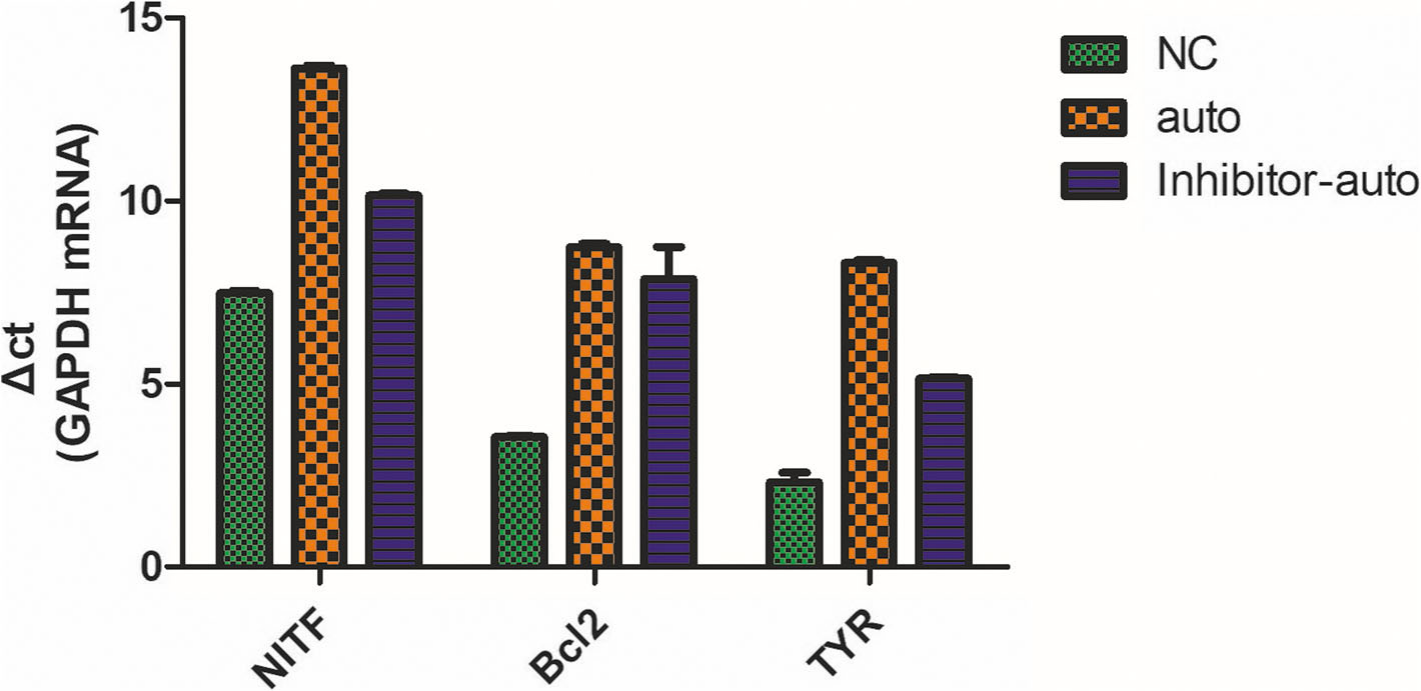
Analysis of expression of gene NITF, Bcl2, and TYR by RT-PCR (mean ± SD, *n* = 3)

The expression of NITF, Bcl2, and TYR were higher in the auto group and inhibitor-auto group than the normal group. Among them, the expression of NITF, Bcl2, and TYR mRNA in the auto group was higher than that of the inhibitor-auto group (Fig. 4).

### Apoptosis was detected using flow cytometry

**Fig. 5:**
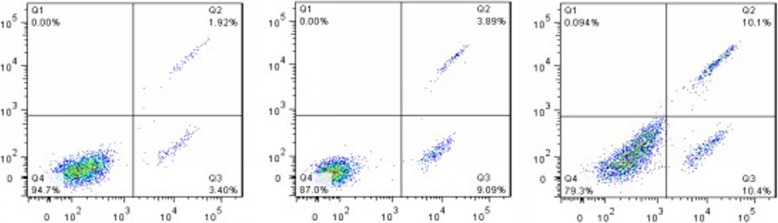
Analysis of chondrocyte apoptosis by flow cytometry. The number of cell apoptosis in Q2 and Q3 areas in the inhibitor-auto group was more than that in the auto group and normal group. NC, normal group

The number of cell apoptosis in the inhibitor-auto group was more than that in the auto group (Fig. 5), which indicated that autophagy could inhibit apoptosis and promote cell growth.

## Discussion

Autophagy is an evolutionary process in which cell proteins and organelles are engulfed by autophagosomes and eventually transferred to lysosomes for degradation. Autophagy includes three types of autophagy in mammals: macroautophagy, microautophagy, and chaperone-mediated autophagy. Autophagy is one of the most studies consisting of two stages: (1) the formation of phagophore and isolation membrane, which means the formation of autophagosomes, and (2) maturation and degradation stages, autophagosomes, and lysosomes combine to form a double body [11]. At present, most researches on autophagy focus on the early stage of autophagy, and most autophagy-related genes are found to regulate the formation of autophagosomes, but relatively few researches on lysosomes in autophagy.

TFEB is considered the main activating agent for lysosomal autophagy gene transcription [6]. Currently, the number of studies on TFEB-mediated autophagy regulation is increasing. It had been reported that TFEB overexpression in endothelial cells in mice had less atherosclerotic lesions than the control group. It suggested that TFEB inhibited inflammation in endothelial cells and reduced the occurrence of atherosclerosis [12]. Overexpression of TFEB in endothelial cells reduced the concentration of ROS and increased the expression of antioxidant genes HO1 and superoxide dismutase SOD2, achieving the effect of anti-oxidative stress [13, 14]. TFEB was also involved in tumor pathogenesis. For example, the tumor suppressor p53 could regulate the nuclear translocation and activity of TFEB in lung cancer cells. Deletion or pifithrin-α chemical inhibition of p53 could promote TFEB to transfer from cytoplasm to nucleus, thereby increasing the synthesis of lysosomes and autophagosomes in lung cancer cells mediated by TFEB [15].

Bcl-2 gene, namely B cell lymphoma/leukemia-2 gene, has the effect of inhibiting apoptosis. The expression of Bcl-2 is reduced, leading to serious apoptosis [16]. Autophagy can inhibit apoptosis and promote cell survival. There are many binding sites on the promoter of the kinase gene (Tyr), among which is the Mitf gene. Tyr is involved in biosynthesis and transportation through the regulation of the Mitf gene. It had been reported in the literature that Mitf gene was involved in the directed development of cells and regulated the proliferation and apoptosis of melanocytes [17]. Meanwhile, Bcl-2 gene is also regulated by Mitf, and NITF, Bcl2, and TYR jointly regulate the survival and development of chondrocytes.

Autophagy was progressive and played an important role in regulating the signaling pathways that promoted survival and death of cells. Autophagy as a protective mechanism could protect cells from apoptosis and necrosis and facilitate cell survival [18].

**Fig. 6:**
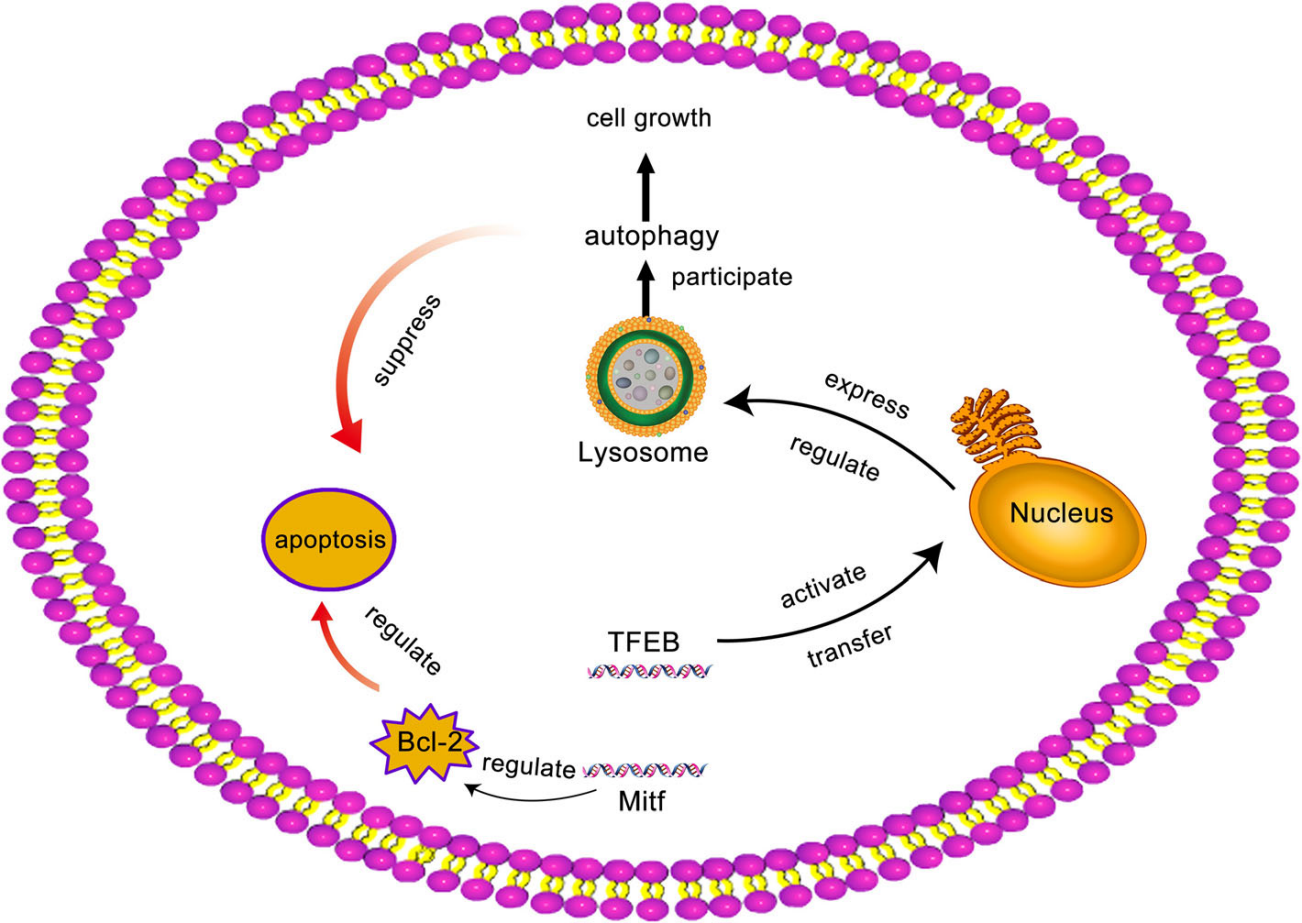
Bcl-2 gene was also regulated by Mitf, regulating the survival and development of chondrocytes

In a word, autophagy damaged proteins in the cytoplasm or damaged organelles into vesicles, and fusion with lysosomes form autophagy lysosome. It degraded the contents of the package and provided nutrition or cell recycling raw material supply, in order to realize the need of cell metabolism and organelles update [19]. TFEB is a major transcriptional regulator of autophagy and lysosome biosynthesis. TFEB is isolated in the cytoplasm and transferred to the nucleus once activated according to lysosome expression and regulation, promoting lysosome synthesis and participating in autophagy [20, 21]. The TFEB gene was regulated by the Mitf gene, inhibiting cell apoptosis and regulating cell proliferation and apoptosis [22]. Meanwhile, Bcl-2 gene was also regulated by Mitf, regulating the survival and development of chondrocytes (Fig. 6).

## Conclusion

Autophagy can inhibit apoptosis, promote chondrocyte growth and bone regeneration, and restore knee joint injury of rats. The main mechanism is to promote the effect of TFEB-mediated lysosomal autophagy.

## Data Availability

The data and materials in the research can be obtained if necessary.

## Abbreviations

3-MA: 3-Methyladenine
ANOVA: Analysis of variance
SD: Standard deviation
TFEB: Transcription factor EB
